# Identifying Potential miRNA Biomarkers for Gastric Cancer Diagnosis Using Machine Learning Variable Selection Approach

**DOI:** 10.3389/fgene.2021.779455

**Published:** 2022-01-10

**Authors:** Neda Gilani, Reza Arabi Belaghi, Younes Aftabi, Elnaz Faramarzi, Tuba Edgünlü, Mohammad Hossein Somi

**Affiliations:** ^1^ Department of Statistics and Epidemiology, Faculty of Health, Tabriz University of Medical Sciences, Tabriz, Iran; ^2^ Department of Mathematics, Uppsala University, Uppsala, Sweden; ^3^ Department of Statistics, Faculty of Mathematical Science, University of Tabriz, Tabriz, Iran; ^4^ Tuberculosis and Lung Diseases Research Center, Tabriz University of Medical Sciences, Tabriz, Iran; ^5^ Liver and Gastrointestinal Diseases Research Center, Tabriz University of Medical Sciences, Tabriz, Iran; ^6^ Department of Medical Biology, Faculty of Medicine, Muğla Sıtkı Koçman University, Muğla, Turkey

**Keywords:** miRNA, machine learning, boruta algorithm, gastric cancer, hsa-miR-1343-3p, AUC, GSE106817, GSE113486

## Abstract

**Aim:** This study aimed to accurately identification of potential miRNAs for gastric cancer (GC) diagnosis at the early stages of the disease.

**Methods:** We used GSE106817 data with 2,566 miRNAs to train the machine learning models. We used the Boruta machine learning variable selection approach to identify the strong miRNAs associated with GC in the training sample. We then validated the prediction models in the independent sample GSE113486 data. Finally, an ontological analysis was done on identified miRNAs to eliciting the relevant relationships.

**Results:** Of those 2,874 patients in the training the model, there were 115 (4%) patients with GC. Boruta identified 30 miRNAs as potential biomarkers for GC diagnosis and hsa-miR-1343-3p was at the highest ranking. All of the machine learning algorithms showed that using hsa-miR-1343-3p as a biomarker, GC can be predicted with very high precision (AUC; 100%, sensitivity; 100%, specificity; 100% ROC; 100%, Kappa; 100) using with the cut-off point of 8.2 for hsa-miR-1343-3p. Also, ontological analysis of 30 identified miRNAs approved their strong relationship with cancer associated genes and molecular events.

**Conclusion:** The hsa-miR-1343-3p could be introduced as a valuable target for studies on the GC diagnosis using reliable biomarkers.

## Introduction

Gastric cancer (GC) is a significant global health issue due to being the fifth leading cancer worldwide as well as the third cancer-related death leading cause, which leads to nearly 8,00,000 deaths annually (Bray, 2018). Morbidity and mortality due to GC have reduced in recent years, though the rate of 5-year survival is still fairly low (Howlader, 2014). A significant prognostic factor is the stage of cancer at the diagnosis time. The 5-year survival of GC patients is below 30% if the disease is diagnosed at the advanced stages (Hundahl et al., 2000), while the 5-year survival of patients ranges between 70 and 90% if diagnosed at the early stages (Choi, 2015). Thus, GC will remain among the toughest challenges for physicians and researchers for so long since GC is not symptomatic until the advanced stages; this is why effective screening approaches for the early detection of GC are mandatory to overcome GC mortalities (Penon et al., 2014). Presently, gastroscopy is yet the standard test to diagnose GC (Veitch et al., 2015). Nonetheless, this screening approach is invasive and costly. Furthermore, minimally invasive or non-invasive markers, including carcinoembryonic antigen (CEA) and carbohydrate antigen 19-9 (CA19-9) have been commonly used clinically, though these markers are neither specific nor sensitive enough for GC early diagnosis (Carpelan-Holmström et al., 2002). Due to non-specific symptoms and the absence of an early diagnosis, a great number of patients with GC are diagnosed at the advanced stages (Hundahl et al., 2000; Hartgrink et al., 2009). Thus, cost-effective and non-invasive biomarkers are immediately required for the early diagnosis of GC.

Recent genome analysis revealed several biomarkers which are related to RNA, DNA, exosome, et cetera. A class of endogenous non-coding RNAs is MicroRNAs (miRNAs) (nearly 22 nt) which module the expression of the gene after transcription through degradation or translation blockage of target mRNAs (Bartel, 2004; Caldas and Brenton, 2005). It is well-known that cancer cells may release miRNAs via exosomes to enhance proliferation and migration (Li, 2018; Yoshimura, 2018; Zeng, 2018). The exosomal miRNAs released into biofluids, including serum, plasma, tear, urine, and gastric juice, may escape being degraded by RNases (Gilad, 2008). Moreover, miRNAs have been suggested as potential biomarkers which may be used to diagnose several types of cancers, including testicular germ cell tumors (using miRNA-371a-3p: specificity 94.0% and sensitivity 90.1%) (Dieckmann, 2019), bladder cancer (using 7-miRNA panel: specificity 87% and sensitivity 95%) (Usuba, 2019a), and hepatocellular carcinoma (using miR-424: specificity 87.13% and sensitivity 95.12%) (Lin, 2015), and lung cancer (Aftabi, 2021). Moreover, several studies reported that numerous miRNAs might be potentially used as biomarkers for GC diagnosis (Zhou, 2010; Cui, 2013; Su, 2014). Nonetheless, most of the miRNA biomarkers are not developed using comprehensive data mining according to miRNA profiling, and even they lack proper external efficacy validation (Link and Kupcinskas, 2018; Wei, 2019). Instead, recently, Artificial intelligence Technology (AT) usage in the field of microarray Data has attracted more attention. The disadvantage of the conventional statistical models, including logistic regression, was that they excluded the possible interaction terms and highly correlated variables; thus, they might lose a part of useful information, which might decrease their accuracy, specifically in the case of high dimensional miRNA data analysis (Alpaydin, 2020). Furthermore, the traditional models are not able to capture variables’ non-linear associations (James et al., 2013; Gilani et al., 2017; Gilani et al., 2019). Instead, Machine Learning (ML) is able to deal with non-linear structures as well as detecting all the possible interactions which may exist between predictors (Gilani et al., 2018; Wiemken and Kelley, 2020).

Machine learning has several algorithms of which the decision trees (DT), random forests (RF), extreme gradient boosted trees (XGBT), and artificial neural networks (ANN) that have been frequently applied in medicine (Cleophas and Zwinderman, 2015; Deo, 2015), particularly in prediction of cancer (DeGregory, 2018; Fakhari et al., 2019). Random forest is a tree-based classification algorithm, and as the name indicates, the algorithm creates a forest with a huge number of trees. It is an ensemble algorithm that combines multiple algorithms. The random forest creates a set of decision trees from a random sample of the training set. It repeats the process with multiple random samples and makes a final decision based on majority voting (Zhou, 2012). Briefly, gradient boosted trees combine multiple classification trees into an additively weighted classifier. Boosting refers to the method where sequentially ascertained trees were trained, meaning each observation was weighted by its error obtained by minimizing the appropriate loss of function in the previous iteration. In this way, boosting is a gradient descent algorithm (Christensen and Bastien, 2016) and forces the classifier to focus on aspects of the data that are difficult to learn (Hastie et al., 2009).

Artificial neural networks have been broadly used in medical studies (Darsey et al., 2015; DeGregory, 2018; Shahid et al., 2019). Such models perform satisfactorily, especially for classification problems with complex and non-linear associations between variables (Hastie et al., 2009). Briefly, artificial neural networks are based on a collection of artificial neurons, which receive and process inputs (predictors), transmit them to other artificial neurons, and produce an output (Zhou, 2012).

Considering the important role of GC early diagnosis in patient’s survival rate and the lack of published article on identifying potential miRNAs for GC prediction at an early stage by AT, the present study aims to identify the potential miRNA for predicting GC by AT in the datasets of Gene Expression Omnibus (GEO) specifically with the stat of the art machine learning models. Traditional statistical models such as linear models previously has been used in looking for GC biomarkers and identified miRNAs with the potential prediction power (Yao, 2020), however, they have not implemented advanced methods such as machine learning and new variable selection approaches such as Synthetic Minority *Oversampling* Technique (SMOTE). In the present study, for the first time, we aimed to use those new techniques for identification of GC related miRNAs with a reliable cut-of and highest possible accurecy in the external validation.

## Methods

### The Applied Datasets

For training sample, we used GSE106817 dataset that is available at https://www.ncbi.nlm.nih.gov/geo/. The dataset consist of the data of 2,566 miRNAs obtained from 2,759 non-cancer controls, and 115 GC cases (4%). In the original study the serum samples of cancer cases and non-cancer controls have been analyzed by microarray for miRNA expression profiles (Yokoi, 2018). For test sample we used GSE113486 dataset, which includes data of miRNA expression profiles from the serum samples of 40 GC cases (28.6%) and 100 normal controls (71.4%) (Usuba, 2019b). All the datasets were serum miRNA profiles based on the same microarray platform, 3D-Gene Huma miRNA V21_1.0.0 (39). The study was approved by the NCCH Institutional Review Board (2015-376, 2016-29) and the Research Ethics Committee of Medical Corporation Shintokai Yokohama Minoru Clinic (6019-18-3772). Written informed consent was obtained from each participant (42). This study was approved by the Ethics Committee of Tabriz University of Medical Sciences (No: IR. TBZMED.REC.1400.006).

### Boruta Machine Learning Algorithm

We used the Boruta machine learning algorithm to select the most critical miRNAs related to GC in the training sample that produce the highest prediction accuracy. In short, Boruta selects the variables that have a high impact on the prediction accuracy by providing the “variable importance” (Kursa and Rudnicki, 2018). We used SMOTE random oversampling to balance the outcome in the GSE106817 data. We then used five-fold cross-validation to find the optimal hyper parameters on DT, RF, LR, XGBT, and ANN to choose the best approaches in the balanced sample using the most important variables selected by Boruta. Once the prediction models were developed, we applied them on the test sample GSE113486 to verify the accuracy of developed prediction approach. We looked for an algorithm that may generate a higher predictive power among the 5 ML algorithms in terms of the yielded areas under the ROC curves (AUCs). Sensitivity, specificity, positive predictive value, negative predictive value, misclassification rate, and Kappa were assessed. The guidelines of developing transparent multivariable prediction models was followed for these analysis (Moons, 2015).

### GeneCodis Ontological Analysis

GeneCodis is a web-based tool for the ontological analysis of lists of genes, proteins, and regulatory elements like miRNAs, transcription factors, and CpGs. It can be used to determine biological annotations or combinations of annotations that are significantly associated to a list of genes under study with respect to a reference list. As well as single annotations, this tool allows users to simultaneously evaluate annotations from different sources, for example GO Biological Process and KEGG. To this end, and before computing *p*-values, it uses the apriori algorithm to extract sets of annotations that frequently co-occur in the analyzed list of genes (Garcia-Moreno, 2021). We used GeneCodis 4 (https://genecodis.genyo.es/) for ontological analysis of the identified miRNAs list.

## Results

Of those 2,874 patients included in this study, there were 115 (4%) patients with gastric cancer. This analysis consists of 2,566 miRNAs.

### Selected miRNAs as Potential GC Biomarkers

Of those 2,566 miRNA in GSE106817 data, the Boruta algorithm initially selected 108 miRNA using Gini Index measurement (results are not shown here). The processing time was 17.24 minuts There were 77 tentative variables at the first stage. After fixing the tentative features, Boruta identified 156 miRNA for the analysis (results are not shown here). The process took 99 iterations convergence. It was observed that hsa-miR-1343-3p had the highest importance for prediction accurecy (minimum importance; 6.47, median importance; 11.44, mean importance; 10.81; maximum importance; 13.63) among all identified miRNAs. The hsa-miR-1290 and hsa-miR-5100 had the second and third highest importance, with mean importance of 8.69 and 8.66, respectively (Table 1).

**TABLE 1:** Selected important miRNAs by Boruta Algorithm Using XGboost Algorithm.

No	miRNA	Importance	Se (%)	Sp (%)	PPV (%)	NPV (%)	AUC (%)	Accuracy (%)	Kappa (%)
1	hsa-miR-1343-3p	100.00	100.00	100.00	100.00	100.00	100.00	100.00	1.00
2	hsa-miR-1290	80.39	92.50	98.00	94.87	97.03	99.05	96.43	0.96
3	hsa-miR-5100	80.11	100.00	99.00	97.56	100.00	99.23	99.29	0.99
4	hsa-miR-6746-5p	64.57	100.00	93.00	85.11	100.00	97.23	95.00	0.95
5	hsa-miR-4532	64.85	67.50	100.00	100.00	88.50	95.11	90.71	0.91
6	hsa-miR-8073	61.79	97.50	100.00	100.00	99.01	100.00	99.29	0.99
7	hsa-miR-1228-5p	56.24	97.50	100.00	100.00	99.01	100.00	99.29	0.99
8	hsa-miR-1199-5p	54.12	62.50	97.00	89.29	86.61	92.56	87.14	0.87
9	hsa-miR-3622a-5p	54.49	80.00	99.00	96.97	92.52	97.26	93.57	0.94
10	hsa-miR-8060	53.75	85.00	98.00	94.44	94.23	98.79	94.29	0.94
11	hsa-miR-1246	50.42	92.50	100.00	100.00	97.09	99.90	97.86	0.98
12	hsa-miR-4787-3p	50.32	90.00	100.00	100.00	96.15	98.75	97.14	0.97
13	hsa-miR-6087	49.68	22.50	88.00	42.86	73.95	62.70	69.29	0.69
14	hsa-miR-4259	47.55	90.00	98.00	94.74	96.08	99.04	95.71	0.96
15	hsa-miR-6877-5p	46.90	92.50	94.00	86.05	96.91	97.73	93.57	0.94
16	hsa-miR-124-3p	45.42	92.50	94.00	86.05	96.91	96.81	93.57	0.94
17	hsa-miR-6787-5p	45.14	87.50	99.00	97.22	95.19	99.70	95.71	0.96
18	hsa-miR-4454	45.05	95.00	98.00	95.00	98.00	98.10	97.14	0.97
19	hsa-miR-6760-5p	45.42	90.00	94.00	85.71	95.92	98.58	92.86	0.93
20	hsa-miR-668-5p	45.24	77.50	98.00	93.94	91.59	96.44	92.14	0.92
21	hsa-miR-6762-5p	42.09	45.00	92.00	69.23	80.70	88.94	78.57	0.79
22	hsa-miR-3191-3p	40.43	75.00	94.00	83.33	90.38	93.48	88.57	0.89
23	hsa-miR-1268b	39.32	70.00	94.00	82.35	88.68	93.91	87.14	0.87
24	hsa-miR-1185-2-3p	39.13	30.00	87.00	48.00	75.65	53.88	70.71	0.71
25	hsa-miR-6131	38.30	87.50	98.00	94.59	95.15	99.21	95.00	0.95
26	hsa-miR-920	38.39	87.50	96.00	89.74	95.05	98.26	93.57	0.94
27	hsa-miR-4635	38.02	77.50	98.00	93.94	91.59	95.38	92.14	0.92
28	hsa-miR-6724-5p	37.28	45.00	81.00	48.65	78.64	74.35	70.71	0.71
29	hsa-miR-1185-1-3p	37.19	20.00	85.00	34.78	72.65	54.70	66.43	0.66
30	hsa-miR-422a	38.02	55.00	87.00	62.86	82.86	72.94	77.86	0.78

The balanced training data using SMOTE random oversampling technique had 1,376 cancer cases and 1,498 non-cancer controls. We trained DT, RF, LR, XGBT, and ANN perdition models with the selected miRNAs in the balanced training data.

### Prediction Models and Accuracy in the Validation Data

**FIGURE 1:**
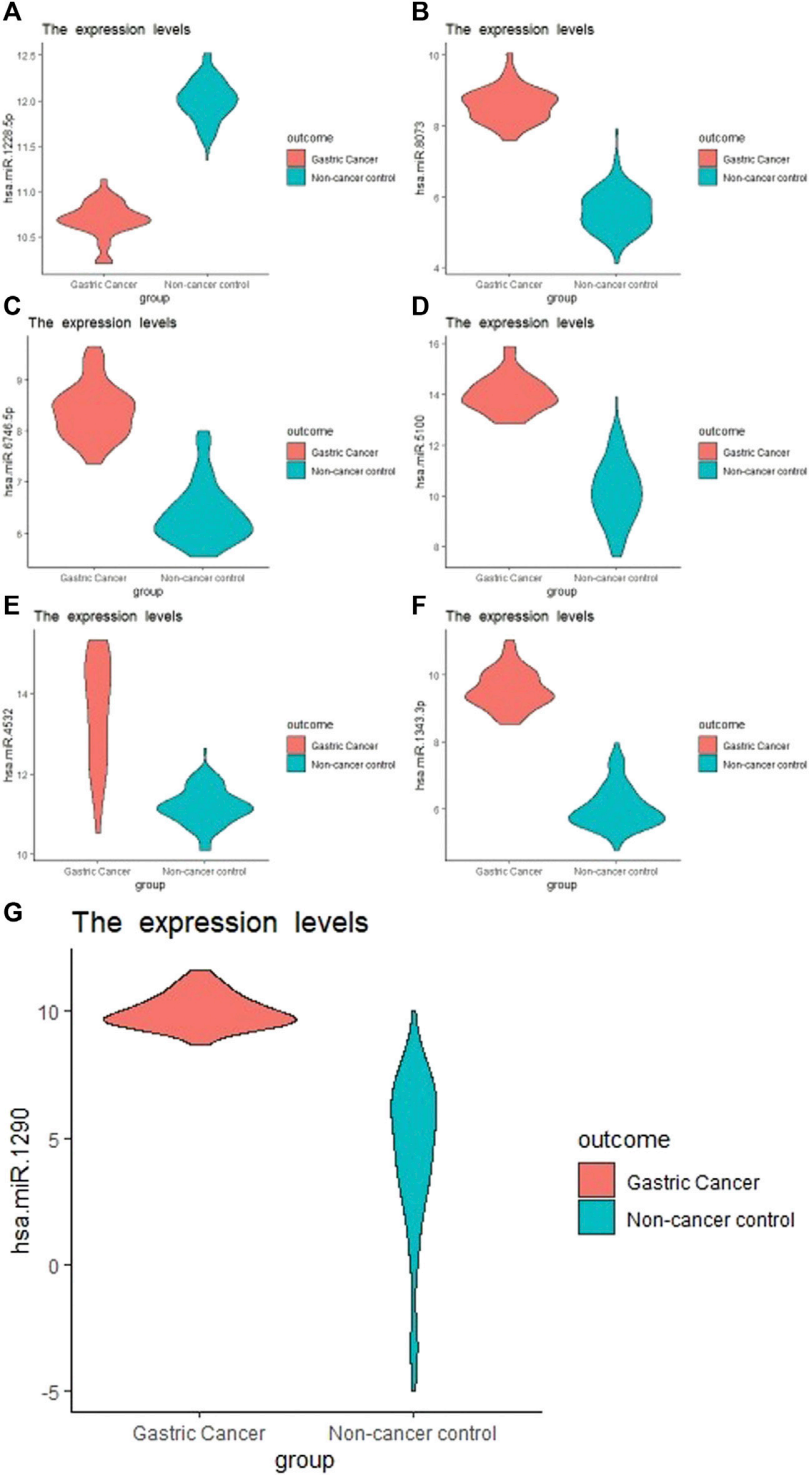
Boxplot of the selected miRNA from Boruta Algorithm. **(A)**, hsa-miR-1228-5p; **(B)**, hsa-miR-8073; **(C)**, hsa-miR-6746-5p; **(D)**, hsa-miR-5100; **(E)**, hsa-miR-4532; **(F)**: hsa-miR-1343-3p; **(G)**, hsa-miR-1290.

The external validation data GSE113486 had 40 (28.6%) gastric cancer and 100 (71.4%) non-cancer (controls). hsa-miR-1343-3 produced the highest prediction accuracy for GC prediction (Table 1). For the hsa-miR-1343-3, all of the accuracy measures including AUC, sensitivity and specificity, positive predictive value, negative predictive value, Kappa were 100%. According to the decision trees, the cut-off point for this miRNA was 8.2 (Figure 1). Further, hsa-miR-8073 and hsa-miR-1228-5p produced 100% AUC but other accuracy measures were not 100%. On the other hsa-miR-1185-1-3p had the lowest AUC which has the least contribution to the prediction of GC.

**FIGURE 2:**
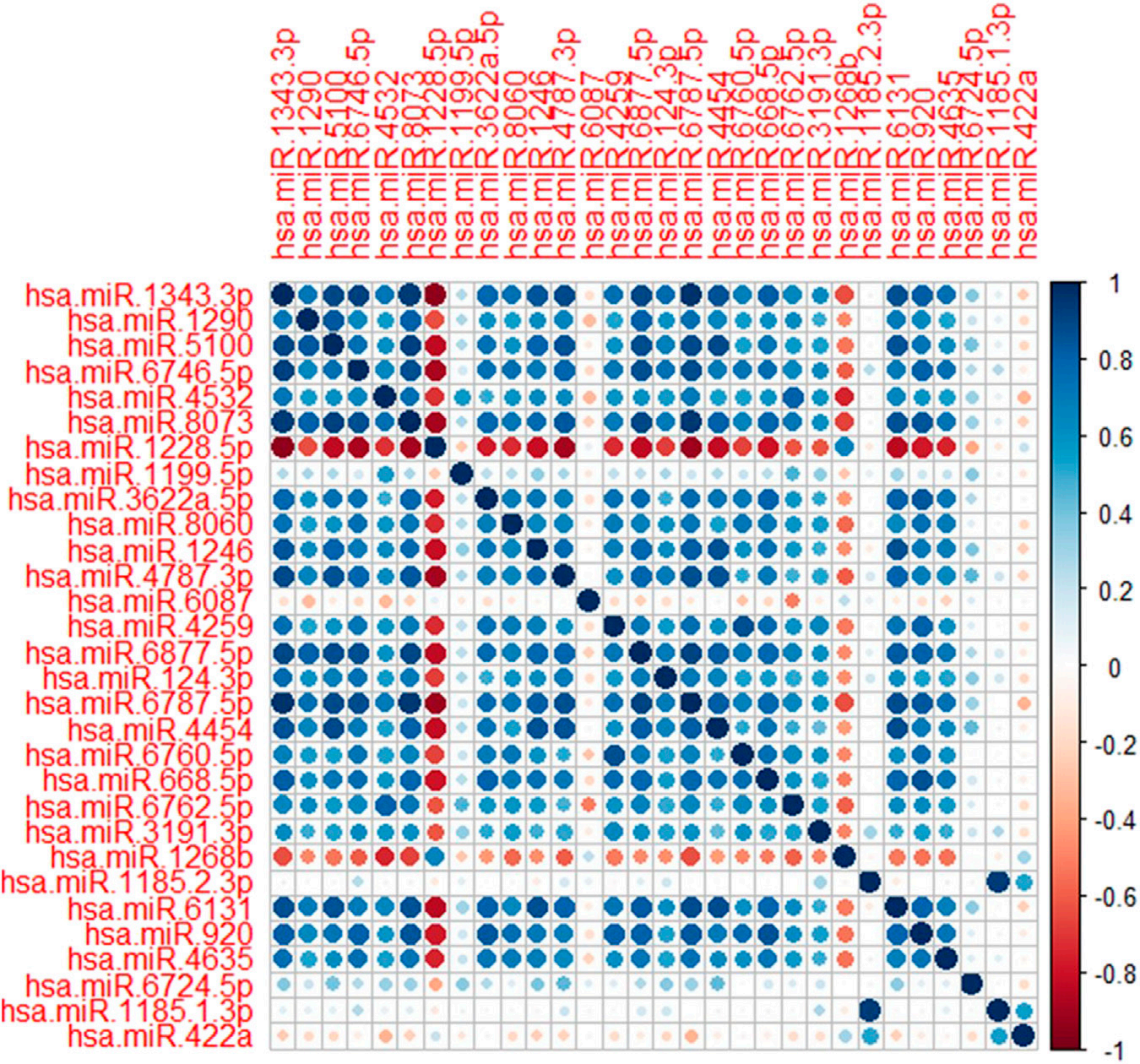
Correlation plot of the selected miRNAs. Dark blue and dark red shows the strength of the correlations between miRNAs.

**FIGURE 3:**
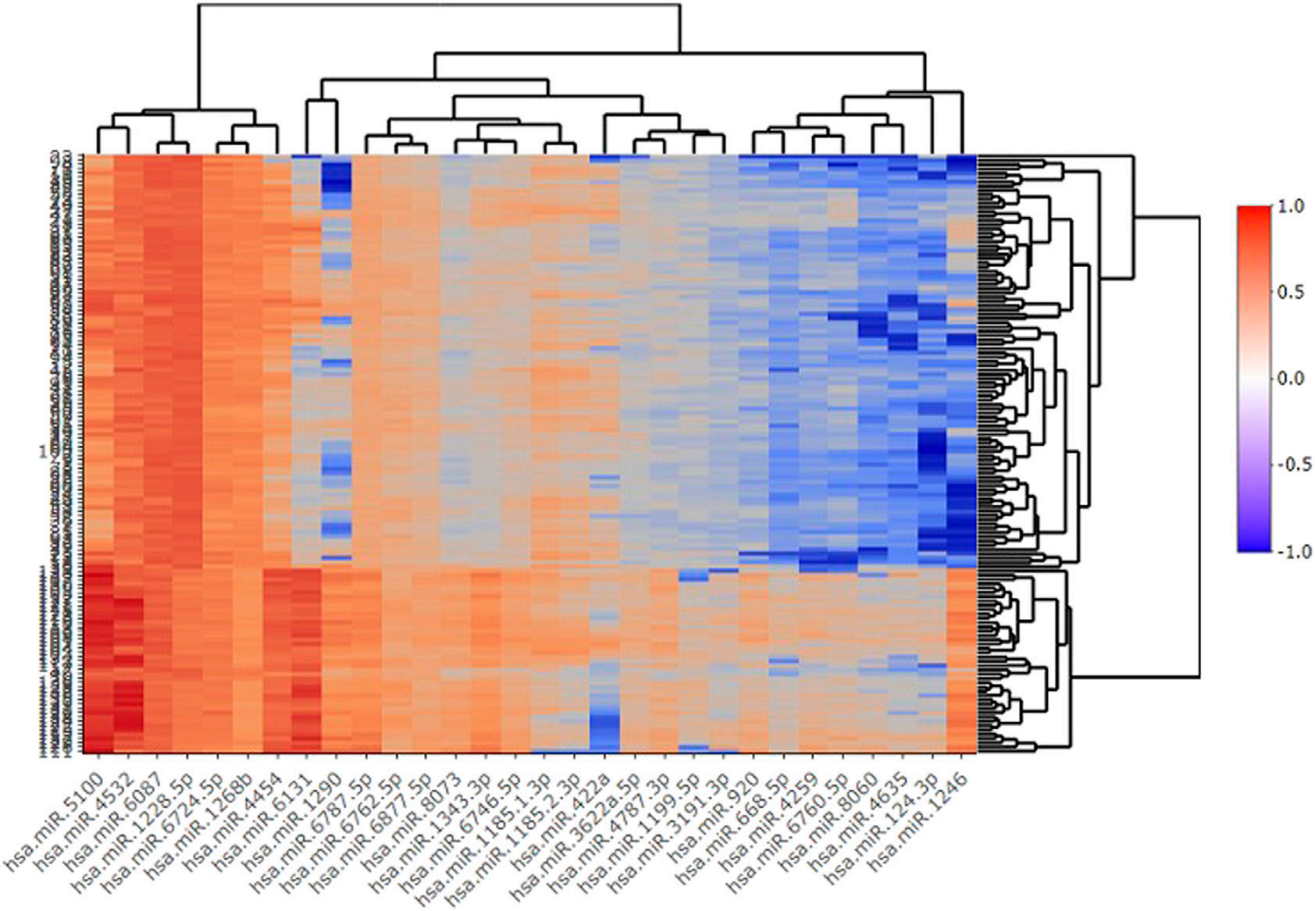
Heathmap plot of clustering of 30 selected miRNAs.

Among several models discussed in the study, the XGBT algorithm had better prediction accuracy overall (Table S1-S4). However, for hsa-miR-1343-3 all models had consistently 100% accuracy which indicates that this miRNA may strongly predict GC. For some miRNA such as hsa-miR-422a XGBT algorithm could predict GC with higher accuracy than the logistic regression and decision trees. Figure 2 shows the correlation of the important miRNAs. It can be observed that most of the identified miRNAs except hsa-miR-422a, hsa-miR-1185-1-3p, hsa-miR-1185-2-3p, hsa-miR-6087, and hsa-miR-1199-5p are highly correlated. Consequently, clustering of correlated those miRNAs is helpful for the identification of cancerous and non-cancerous patients. Finally, Heatmap plot indicates the result of the hierarchical clustering analysis of the 30 selected miRNAs, which represents that identified miRNAs can easily distinguish GC cases and controls in test sample obtained from GSE113486 dataset (Figure 3).

### Ontological Analysis

**FIGURE 4:**
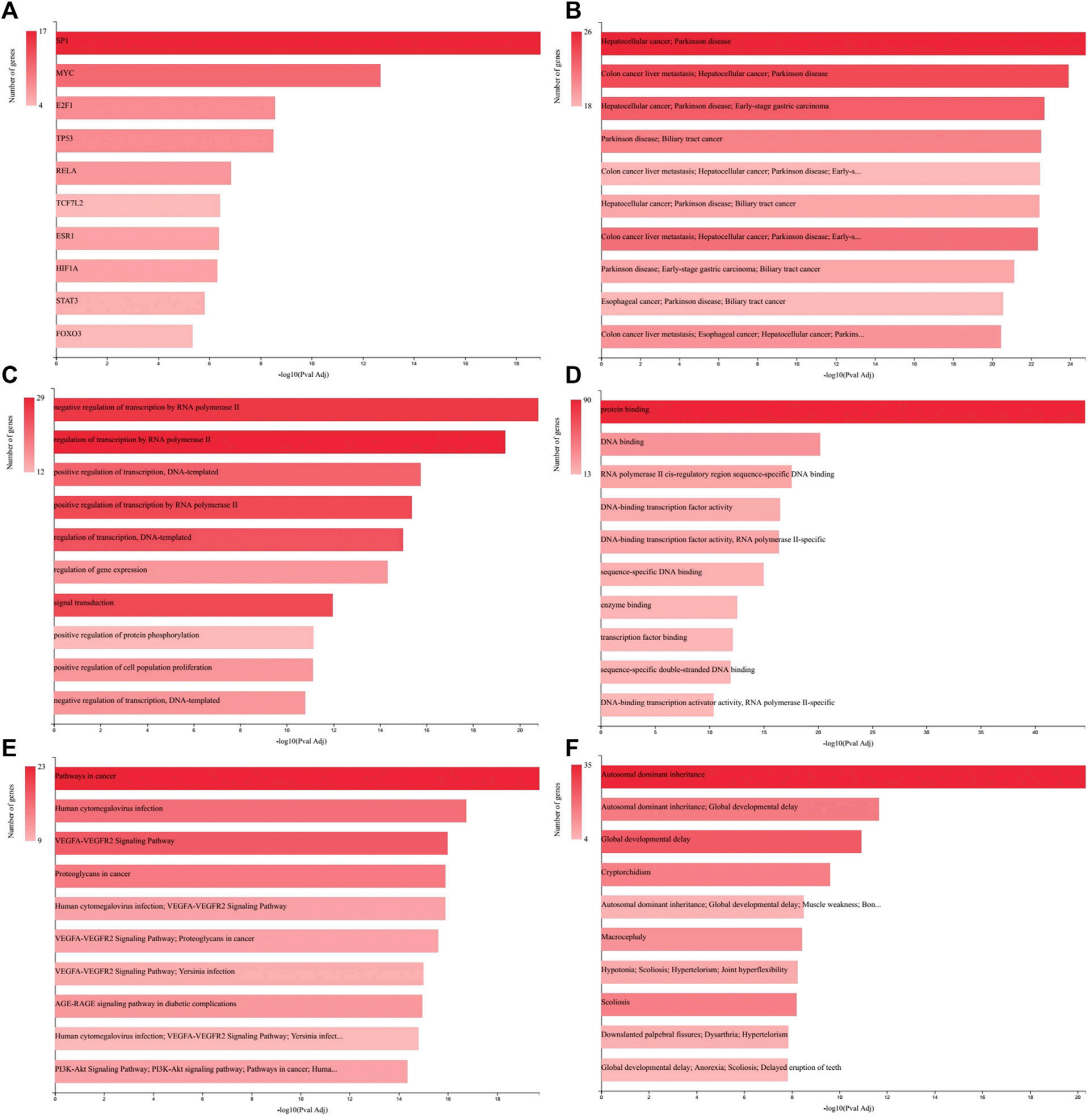
GeneCodis Ontological analysis. Visualizations generated for 10 top terms of related categories with our identified miRNAs list are presented here for Transcription Factors **(A)**, Co-annotation of miRNAs-based analysis using HMDD v3, MNDR, and TAM2 **(B)**, GO Biological Process **(C)**, GO Molecular Function **(D)**, Co-annotation of KEGG Pathways, Panther Pathways, and WikiPathways databases **(E)**, and Co-annotation of HPO and OMIM databases **(F)**.

Regulatory, functional, and perturbation analysis by GeneCodis 4 showed that 30 identified miRNAs (Table 1) are related strongly to the cancer-associated genes and molecular events (Figure 4). Visualizations generated for 10 top terms of associations with Transcription Factors (Figure 4A), co-annotation of HMDD v3 (the Human microRNA disease database), MNDR (Mammalian ncRNA-Disease Repository), and TAM2 (The tool for annotations of human miRNAs) databases (Figure 4B), GO (Gene Ontology and GO Annotations) Biological Process (Figure 4C), GO Molecular Function (Figure 4D), co-annotation of KEGG (Kyoto Encyclopedia of Genes and Genomes) Pathways, Panther (Protein ANalysis THrough Evolutionary Relationships) Pathways, and WikiPathways (Figure 4E), and co-annotation of HPO (The Human Phenotype Ontology) and OMIM (Online Mendelian Inheritance in Man) databases (Figure 4F).

## Discussion

Using artificial intelligence technology, we identified hsa-miR-1343-3 as a very strong nominate for biomarker analysis of GC diagnosis. The value of hsa-miR-1343-3 higher than 8.2 indicates that it could be a strong predictor for GC (100% of AUC, 100% of Sensitivity and Sepecificity). We also found three other miRNAs (hsa-miR-8073 and hsa-miR-1228-5p) with a great contribution to the GC prediction. A medical expert can use these findings for the early detection of GC instead of using costly and time-consuming tools such as colonoscopy Yao et *al.* (Yao, 2020).

This study had several strengths compared to the previous studies. Compared to Shi et al. that identified the miR-1246 as the potential biomarker of GC that generated the AUC of 83%, our study identified the hsa-miR-1343-3p using the Boruta algorithm that led to a significant increase in the AUC (Shi and Zhang, 2019). The study of Yao et al., selected three miRNAs that produced similar precision to our study that using only single miRNA that may have economical merits. Further, their study used a limited sample size (70 gastric cancer patients and 374 non-cancer controls) in the training set that may lead to an inferior model. The current study used very advanced variable selection methods and the state of the art machine learning approaches that produced consistent results. Another merit of the study is introducing a simple cut-off point of 8.2 using decision trees that may has very practical value in GC classification.

Figure 4A depicted that among the transcription factors related to the genes associated with the identified miRNAs list (Table 1) the SP1, MYC, and E2F1 have higher priorities. SP1 protein expression is up regulated in GC tissues compared with normal tissues and is positively associated with depth of invasion and TNM stage of GC (Shi and Zhang, 2019). MYC is an oncogene responsible for excessive cell growth in cancer, enabling transcriptional activation of genes involved in cell cycle regulation, metabolism, and apoptosis, and is usually overexpressed in GC (Maués, 2018). E2F1 is a member of the E2F family that functions in cell cycle progression and apoptosis induction in response to DNA damage. Deregulated E2F1 acts as a driving force in GC progression and promotes tumor invasion and metastasis independently from its other cellular activities (Yan, 2014).

As depicted in Figure 4B gastrointestinal cancers including hepatocellular cancer, colon cancer, biliary tract cancer, and especially early-stage GC are among the most related diseases to the analyzed miRNAs list. From biological process and molecular function perspectives as showed in Figures 4C,D, the regulation of transcription and gene expression, and protein and DNA binding are the most targeted aspects, which are the general aspects of molecular biology of GC (Cervantes et al., 2007; Vauhkonen et al., 2006; Tan and Yeoh, 2015). Co-annotation of three pathway databases (Figure 4E) has shown that the miRNAs list is general in relation with pathways in cancer, VEGFA-VEGFR2 signaling pathway and PI3K-Akt signaling pathway. The increased expression of VEGFA in the tubular glands and VEGFR2 in the endothelium of GC samples mainly in the T2, T3 and T4 stages of tumor progression has been reported previously (Tamma, 2018). Also, it is showed that the PI3K/AKT/mTOR pathway is activated in GC with overexpression in tumor tissue, which is correlated with the depth of tumor infiltration and the presence of lymph node metastases (Tapia, 2014). Surprisingly, relation with Human cytomegalovirus infection, which was identified in our pathway analysis, has been reported to be associated with the development of GC (Jin, 2014) and GC lymphatic metastasis (Zhang, 2017).

The analysis of human phenotype and Mendelian inheritance ontologies identified Autosomal dominant inheritance and Global developmental delay among the most related phenomena with our miRNAs list. It is reported that gastric adenocarcinoma and proximal polyposis of the stomach is an autosomal dominant syndrome (Worthley, 2012). Also, some common variants have been described for GC and developmental delay (Hansford, 2015; Zhang, 2020).

In our study, we have shown theoreticaly that ther is a strong relationship between hsa-miR-1343-3p and GC. Hsa-miR-1343-3p has been indicated as a tumor suppressor for many types of cancer. It has been suggested that miR-1343-3p, which regulates the oncogenic effect of TEA domain transcription factors is associated with GC (Zhou, 2017). The correlation between hsa-miR-1343-3p and lung adenocarcinoma was evaluated and its expression was found to be low in patients with vascular invasion (Kim, 2017). Yuan et al. demonstrated that hsa-miR-1343-3p is consistently down-regulated in colon, prostate, and pancreatic cancers. Also, hsa-miR-1343-3p has been proposed as a biomarker to distinguish pancreato-biliary malignancy from non-malignant diseases. The major genes targeted by miR-1343-3p have been identified (Yuan, 2016). In this context, these target genes and their interaction with GC should also be investigated. The hsa-miR-1343-3p targets including SHISA7, TGFBR1, DLGAP3, SPRED1, ATXN7L3, and PLXDC2 genes are listed at MIRDB (http://mirdb.org/). Among them transforming growth factor beta-1 (TGFβ1) play an important role in carcinogenesis upon binding its receptor (TGFBR1). It acts as a tumor suppressor by inhibiting cellular proliferation or by promoting cellular differentiation and apoptosis. However, it turns to be a tumor promoter by stimulating angiogenesis and cell motility, suppressing the immune response, and increasing progressive invasion and metastasis (Yuan, 2016). Other reports have also revealed that hsa-miR-1343-3p reduces the expression of transforming growth factor-β (TGF-β) receptor-1, which induces angiogenesis through vascular endothelial growth factor (VEGF)-mediated apoptosis. Therefore, hsa-miR-1343-3p may also play an anti-angiogenic role (Ferrari et al., 2009; Stolzenburg et al., 2016; Kim, 2017). He et al. determined that TGFBR1 genes’ two polymorphisms (rs334348, rs10512263) were associated with the risk of GC (He, 2018). In another study, Zhang et al. have shown that silencing of TGFBR1 inhibited cell proliferation, migration, invasion, and EMT in GC cells (Zhang, 2019).

Discs large associated proteins (DLGAPs) family has been implicated in psychological and neurological diseases. However, few studies have explored the association between the expression of DLGAPs and different types of cancer. Liu et al. has suggested that the significant overexpression of DLGAP4 in GC may be a promising potential prognostic marker for GC (Liu et al., 2018). Aslo, Liu et al. have determined decreased expression of SPRED1 in GC tissues (Liu et al., 2020).

However, there were certain limitations in our study. We had relatively small sample size in GC group. Other limitations were the pathological information such as the tumor stage, age or other factors which were not available in our datasets. Nonetheless, the prediction accuracy of our model has high enough (100% AUC) for clinical use. Further, we were unable to do the survival analysis to further validate the markers identified in this paper based on public available data (Howlader, 2014).

## Conclusion

Using several state of the art machine learning methods and Boruta algorithm, we identified several miRNAs that can predict GC. Specifically, hsa-miR-1343-3p, which identified by cut-off point of 8.2 may be nominated as a highly reliable biomarker for, GC diagnosis after meticulous empirical tests.

## Data Availability

Publicly available datasets were analyzed in this study. This data can be found here: https://www.ncbi.nlm.nih.gov/geo/.
