# Supplementary material for: Identifying Potential miRNA Biomarkers for Gastric Cancer Diagnosis Using Machine Learning Variable Selection Approach
Source: Front Genet. 2022 Jan 10;12:779455. doi: 10.3389/fgene.2021.779455 (PMC8785967; doi:10.3389/fgene.2021.779455)
Supplement: Supplementary file 1 [file Table1.DOCX]

**Supplementary Materials**

**Table S1.** Performance Measure of logistic regression model with selected miRNAs

| No. | miRNA | Se (%) | Sp (%) | PPV (%) | NPV (%) | AUC (%) | Accuracy (%) | Kappa (%) |
| --- | --- | --- | --- | --- | --- | --- | --- | --- |
| 1 | **hsa-miR-1343-3p** | **100.00** | **100.00** | **100.00** | **100.00** | **100.00** | **100.00** | **100.00** |
| 2 | hsa-miR-1290 | 100.00 | 98.00 | 95.24 | 100.00 | 99.43 | 98.57 | 96.55 |
| 3 | hsa-miR-5100 | 100.00 | 97.00 | 93.02 | 100.00 | 99.53 | 97.86 | 94.87 |
| 4 | hsa-miR-6746-5p | 100.00 | 92.00 | 83.33 | 100.00 | 99.00 | 94.29 | 86.79 |
| 5 | hsa-miR-4532 | 65.00 | 100.00 | 100.00 | 87.72 | 94.13 | 90.00 | 72.63 |
| 6 | hsa-miR-8073 | 100.00 | 99.00 | 97.56 | 100.00 | 99.95 | 99.29 | 98.26 |
| 7 | hsa-miR-1228-5p | 97.50 | 100.00 | 100.00 | 99.01 | 100.00 | 99.29 | 98.24 |
| 8 | hsa-miR-1199-5p | 80.00 | 90.00 | 76.19 | 91.84 | 82.93 | 87.14 | 68.97 |
| 9 | hsa-miR-3622a-5p | 97.50 | 95.00 | 88.64 | 98.96 | 98.84 | 95.71 | 89.81 |
| 10 | hsa-miR-8060 | 95.00 | 98.00 | 95.00 | 98.00 | 98.10 | 97.14 | 93.00 |
| 11 | hsa-miR-1246 | 100.00 | 97.00 | 93.02 | 100.00 | 99.85 | 97.86 | 94.87 |
| 12 | hsa-miR-4787-3p | 97.50 | 100.00 | 100.00 | 99.01 | 99.98 | 99.29 | 98.24 |
| 13 | hsa-miR-6087 | 32.50 | 75.00 | 34.21 | 73.53 | 60.54 | 62.86 | 7.61 |
| 14 | hsa-miR-4259 | 92.50 | 99.00 | 97.37 | 97.06 | 97.00 | 97.14 | 92.89 |
| 15 | hsa-miR-6877-5p | 92.50 | 92.00 | 82.22 | 96.84 | 98.60 | 92.14 | 81.45 |
| 16 | hsa-miR-124-3p | 95.00 | 93.00 | 84.44 | 97.89 | 96.85 | 93.57 | 84.82 |
| 17 | hsa-miR-6787-5p | 100.00 | 99.00 | 97.56 | 100.00 | 99.95 | 99.29 | 98.26 |
| 18 | hsa-miR-4454 | 100.00 | 96.00 | 90.91 | 100.00 | 99.45 | 97.14 | 93.20 |
| 19 | hsa-miR-6760-5p | 97.50 | 90.00 | 79.59 | 98.90 | 96.68 | 92.14 | 81.97 |
| 20 | hsa-miR-668-5p | 95.00 | 95.00 | 88.37 | 97.94 | 97.25 | 95.00 | 88.02 |
| 21 | hsa-miR-6762-5p | 65.00 | 91.00 | 74.29 | 86.67 | 89.44 | 83.57 | 58.18 |
| 22 | hsa-miR-3191-3p | 80.00 | 96.00 | 88.89 | 92.31 | 92.14 | 91.43 | 78.35 |
| 23 | hsa-miR-1268b | 90.00 | 91.00 | 80.00 | 95.79 | 96.13 | 90.71 | 78.07 |
| 24 | hsa-miR-1185-2-3p | 40.00 | 58.00 | 27.59 | 70.73 | 55.95 | 52.86 | -1.76 |
| 25 | hsa-miR-6131 | 100.00 | 96.00 | 90.91 | 100.00 | 99.15 | 97.14 | 93.20 |
| 26 | hsa-miR-920 | 95.00 | 98.00 | 95.00 | 98.00 | 98.95 | 97.14 | 93.00 |
| 27 | hsa-miR-4635 | 90.00 | 96.00 | 90.00 | 96.00 | 98.55 | 94.29 | 86.00 |
| 28 | hsa-miR-6724-5p | 65.00 | 72.00 | 48.15 | 83.72 | 71.45 | 70.00 | 33.48 |
| 29 | hsa-miR-1185-1-3p | 35.00 | 54.00 | 23.33 | 67.50 | 60.68 | 48.57 | -9.57 |
| 30 | hsa-miR-422a | 55.00 | 70.00 | 42.31 | 79.55 | 65.00 | 65.71 | 22.94 |

**Table S2.** Performance Measure of decision trees model with selected miRNAs

| No. | miRNA | Se (%) | Sp (%) | PPV (%) | NPV (%) | AUC (%) | Accuracy (%) | Kappa (%) |
| --- | --- | --- | --- | --- | --- | --- | --- | --- |
| 1 | **hsa-miR-1343-3p** | **100.00** | **100.00** | **100.00** | **100.00** | **100.00** | **100.00** | **100.00** |
| 2 | hsa-miR-1290 | 100.00 | 98.00 | 95.24 | 100.00 | 99.43 | 98.57 | 96.55 |
| 3 | hsa-miR-5100 | 100.00 | 97.00 | 93.02 | 100.00 | 99.53 | 97.86 | 94.87 |
| 4 | hsa-miR-6746-5p | 100.00 | 92.00 | 83.33 | 100.00 | 99.00 | 94.29 | 86.79 |
| 5 | hsa-miR-4532 | 65.00 | 100.00 | 100.00 | 87.72 | 94.13 | 90.00 | 72.63 |
| 6 | hsa-miR-8073 | 100.00 | 99.00 | 97.56 | 100.00 | 99.95 | 99.29 | 98.26 |
| 7 | hsa-miR-1228-5p | 97.50 | 100.00 | 100.00 | 99.01 | 100.00 | 99.29 | 98.24 |
| 8 | hsa-miR-1199-5p | 80.00 | 90.00 | 76.19 | 91.84 | 82.93 | 87.14 | 68.97 |
| 9 | hsa-miR-3622a-5p | 97.50 | 95.00 | 88.64 | 98.96 | 98.84 | 95.71 | 89.81 |
| 10 | hsa-miR-8060 | 95.00 | 98.00 | 95.00 | 98.00 | 98.10 | 97.14 | 93.00 |
| 11 | hsa-miR-1246 | 100.00 | 97.00 | 93.02 | 100.00 | 99.85 | 97.86 | 94.87 |
| 12 | hsa-miR-4787-3p | 97.50 | 100.00 | 100.00 | 99.01 | 99.98 | 99.29 | 98.24 |
| 13 | hsa-miR-6087 | 32.50 | 75.00 | 34.21 | 73.53 | 60.54 | 62.86 | 7.61 |
| 14 | hsa-miR-4259 | 92.50 | 99.00 | 97.37 | 97.06 | 97.00 | 97.14 | 92.89 |
| 15 | hsa-miR-6877-5p | 92.50 | 92.00 | 82.22 | 96.84 | 98.60 | 92.14 | 81.45 |
| 16 | hsa-miR-124-3p | 95.00 | 93.00 | 84.44 | 97.89 | 96.85 | 93.57 | 84.82 |
| 17 | hsa-miR-6787-5p | 100.00 | 99.00 | 97.56 | 100.00 | 99.95 | 99.29 | 98.26 |
| 18 | hsa-miR-4454 | 100.00 | 96.00 | 90.91 | 100.00 | 99.45 | 97.14 | 93.20 |
| 19 | hsa-miR-6760-5p | 97.50 | 90.00 | 79.59 | 98.90 | 96.68 | 92.14 | 81.97 |
| 20 | hsa-miR-668-5p | 95.00 | 95.00 | 88.37 | 97.94 | 97.25 | 95.00 | 88.02 |
| 21 | hsa-miR-6762-5p | 65.00 | 91.00 | 74.29 | 86.67 | 89.44 | 83.57 | 58.18 |
| 22 | hsa-miR-3191-3p | 80.00 | 96.00 | 88.89 | 92.31 | 92.14 | 91.43 | 78.35 |
| 23 | hsa-miR-1268b | 90.00 | 91.00 | 80.00 | 95.79 | 96.13 | 90.71 | 78.07 |
| 24 | hsa-miR-1185-2-3p | 40.00 | 58.00 | 27.59 | 70.73 | 55.95 | 52.86 | -1.76 |
| 25 | hsa-miR-6131 | 100.00 | 96.00 | 90.91 | 100.00 | 99.15 | 97.14 | 93.20 |
| 26 | hsa-miR-920 | 95.00 | 98.00 | 95.00 | 98.00 | 98.95 | 97.14 | 93.00 |
| 27 | hsa-miR-4635 | 90.00 | 96.00 | 90.00 | 96.00 | 98.55 | 94.29 | 86.00 |
| 28 | hsa-miR-6724-5p | 65.00 | 72.00 | 48.15 | 83.72 | 71.45 | 70.00 | 33.48 |
| 29 | hsa-miR-1185-1-3p | 35.00 | 54.00 | 23.33 | 67.50 | 60.68 | 48.57 | -9.57 |
| 30 | hsa-miR-422a | 55.00 | 70.00 | 42.31 | 79.55 | 65.00 | 65.71 | 22.94 |

**Table S3.** Performance Measure of random forest model with selected miRNAs

| No. | miRNA | Se (%) | Sp (%) | PPV (%) | NPV (%) | AUC (%) | Accuracy (%) | Kappa (%) |
| --- | --- | --- | --- | --- | --- | --- | --- | --- |
| 1 | **hsa-miR-1343-3p** | **100.00** | **100.00** | **100.00** | **100.00** | **100.00** | **100.00** | **100.00** |
| 2 | hsa-miR-1290 | 87.50 | 98.00 | 94.59 | 95.15 | 97.65 | 95.00 | 87.47 |
| 3 | hsa-miR-5100 | 92.50 | 99.00 | 97.37 | 97.06 | 99.46 | 97.14 | 92.89 |
| 4 | hsa-miR-6746-5p | 92.50 | 93.00 | 84.09 | 96.88 | 96.15 | 92.86 | 83.01 |
| 5 | hsa-miR-4532 | 60.00 | 100.00 | 100.00 | 86.21 | 87.38 | 88.57 | 68.18 |
| 6 | hsa-miR-8073 | 80.00 | 100.00 | 100.00 | 92.59 | 100.00 | 94.29 | 85.11 |
| 7 | hsa-miR-1228-5p | 77.50 | 100.00 | 100.00 | 91.74 | 98.75 | 93.57 | 83.11 |
| 8 | hsa-miR-1199-5p | 60.00 | 98.00 | 92.31 | 85.96 | 83.69 | 87.14 | 64.80 |
| 9 | hsa-miR-3622a-5p | 70.00 | 99.00 | 96.55 | 89.19 | 97.40 | 90.71 | 75.20 |
| 10 | hsa-miR-8060 | 82.50 | 98.00 | 94.29 | 93.33 | 92.70 | 93.57 | 83.64 |
| 11 | hsa-miR-1246 | 70.00 | 100.00 | 100.00 | 89.29 | 98.68 | 91.43 | 76.92 |
| 12 | hsa-miR-4787-3p | 75.00 | 100.00 | 100.00 | 90.91 | 98.75 | 92.86 | 81.08 |
| 13 | hsa-miR-6087 | 17.50 | 93.00 | 50.00 | 73.81 | 60.55 | 71.43 | 13.04 |
| 14 | hsa-miR-4259 | 75.00 | 99.00 | 96.77 | 90.83 | 95.55 | 92.14 | 79.36 |
| 15 | hsa-miR-6877-5p | 80.00 | 95.00 | 86.49 | 92.23 | 94.19 | 90.71 | 76.73 |
| 16 | hsa-miR-124-3p | 67.50 | 97.00 | 90.00 | 88.18 | 95.91 | 88.57 | 69.73 |
| 17 | hsa-miR-6787-5p | 82.50 | 99.00 | 97.06 | 93.40 | 99.05 | 94.29 | 85.34 |
| 18 | hsa-miR-4454 | 90.00 | 98.00 | 94.74 | 96.08 | 98.06 | 95.71 | 89.34 |
| 19 | hsa-miR-6760-5p | 80.00 | 96.00 | 88.89 | 92.31 | 95.93 | 91.43 | 78.35 |
| 20 | hsa-miR-668-5p | 72.50 | 98.00 | 93.55 | 89.91 | 96.15 | 90.71 | 75.60 |
| 21 | hsa-miR-6762-5p | 40.00 | 95.00 | 76.19 | 79.83 | 79.23 | 79.29 | 40.82 |
| 22 | hsa-miR-3191-3p | 72.50 | 94.00 | 82.86 | 89.52 | 87.89 | 87.86 | 69.09 |
| 23 | hsa-miR-1268b | 62.50 | 96.00 | 86.21 | 86.49 | 87.84 | 86.43 | 63.76 |
| 24 | hsa-miR-1185-2-3p | 22.50 | 91.00 | 50.00 | 74.59 | 56.94 | 71.43 | 16.17 |
| 25 | hsa-miR-6131 | 72.50 | 99.00 | 96.67 | 90.00 | 97.81 | 91.43 | 77.30 |
| 26 | hsa-miR-920 | 85.00 | 96.00 | 89.47 | 94.12 | 91.45 | 92.86 | 82.23 |
| 27 | hsa-miR-4635 | 67.50 | 98.00 | 93.10 | 88.29 | 93.56 | 89.29 | 71.39 |
| 28 | hsa-miR-6724-5p | 30.00 | 86.00 | 46.15 | 75.44 | 65.91 | 70.00 | 17.88 |
| 29 | hsa-miR-1185-1-3p | 17.50 | 89.00 | 38.89 | 72.95 | 52.80 | 68.57 | 7.78 |
| 30 | hsa-miR-422a | 37.50 | 94.00 | 71.43 | 78.99 | 70.65 | 77.86 | 36.73 |

**Table S4.** Performance Measure of artificial neural networks model with selected miRNAs

| No. | miRNA | Se (%) | Sp (%) | PPV (%) | NPV (%) | AUC (%) | Accuracy (%) | Kappa (%) |
| --- | --- | --- | --- | --- | --- | --- | --- | --- |
| 1 | **hsa-miR-1343-3p** | **100.00** | **100.00** | **100.00** | **100.00** | **100.00** | **100.00** | **100.00** |
| 2 | hsa-miR-1290 | 100.00 | 98.00 | 95.24 | 100.00 | 99.43 | 98.57 | 96.55 |
| 3 | hsa-miR-5100 | 100.00 | 97.00 | 93.02 | 100.00 | 99.53 | 97.86 | 94.87 |
| 4 | hsa-miR-6746-5p | 100.00 | 92.00 | 83.33 | 100.00 | 99.00 | 94.29 | 86.79 |
| 5 | hsa-miR-4532 | 67.50 | 100.00 | 100.00 | 88.50 | 94.13 | 90.71 | 74.79 |
| 6 | hsa-miR-8073 | 100.00 | 99.00 | 97.56 | 100.00 | 99.95 | 99.29 | 98.26 |
| 7 | hsa-miR-1228-5p | 97.50 | 100.00 | 100.00 | 99.01 | 100.00 | 99.29 | 98.24 |
| 8 | hsa-miR-1199-5p | 75.00 | 95.00 | 85.71 | 90.48 | 91.98 | 89.29 | 72.73 |
| 9 | hsa-miR-3622a-5p | 97.50 | 95.00 | 88.64 | 98.96 | 98.84 | 95.71 | 89.81 |
| 10 | hsa-miR-8060 | 92.50 | 98.00 | 94.87 | 97.03 | 98.10 | 96.43 | 91.18 |
| 11 | hsa-miR-1246 | 97.50 | 97.00 | 92.86 | 98.98 | 99.85 | 97.14 | 93.10 |
| 12 | hsa-miR-4787-3p | 97.50 | 100.00 | 100.00 | 99.01 | 99.98 | 99.29 | 98.24 |
| 13 | hsa-miR-6087 | 47.50 | 69.00 | 38.00 | 76.67 | 62.44 | 62.86 | 15.35 |
| 14 | hsa-miR-4259 | 92.50 | 99.00 | 97.37 | 97.06 | 99.18 | 97.14 | 92.89 |
| 15 | hsa-miR-6877-5p | 82.50 | 98.00 | 94.29 | 93.33 | 98.60 | 93.57 | 83.64 |
| 16 | hsa-miR-124-3p | 95.00 | 92.00 | 82.61 | 97.87 | 97.21 | 92.86 | 83.25 |
| 17 | hsa-miR-6787-5p | 100.00 | 99.00 | 97.56 | 100.00 | 99.95 | 99.29 | 98.26 |
| 18 | hsa-miR-4454 | 100.00 | 96.00 | 90.91 | 100.00 | 99.45 | 97.14 | 93.20 |
| 19 | hsa-miR-6760-5p | 97.50 | 90.00 | 79.59 | 98.90 | 98.63 | 92.14 | 81.97 |
| 20 | hsa-miR-668-5p | 95.00 | 93.00 | 84.44 | 97.89 | 97.03 | 93.57 | 84.82 |
| 21 | hsa-miR-6762-5p | 65.00 | 90.00 | 72.22 | 86.54 | 89.44 | 82.86 | 56.70 |
| 22 | hsa-miR-3191-3p | 82.50 | 95.00 | 86.84 | 93.14 | 93.94 | 91.43 | 78.68 |
| 23 | hsa-miR-1268b | 90.00 | 91.00 | 80.00 | 95.79 | 96.13 | 90.71 | 78.07 |
| 24 | hsa-miR-1185-2-3p | 27.50 | 68.00 | 25.58 | 70.10 | 55.95 | 56.43 | -4.40 |
| 25 | hsa-miR-6131 | 100.00 | 97.00 | 93.02 | 100.00 | 99.15 | 97.86 | 94.87 |
| 26 | hsa-miR-920 | 95.00 | 98.00 | 95.00 | 98.00 | 98.95 | 97.14 | 93.00 |
| 27 | hsa-miR-4635 | 90.00 | 94.00 | 85.71 | 95.92 | 95.79 | 92.86 | 82.76 |
| 28 | hsa-miR-6724-5p | 75.00 | 62.00 | 44.12 | 86.11 | 71.40 | 65.71 | 30.58 |
| 29 | hsa-miR-1185-1-3p | 37.50 | 54.00 | 24.59 | 68.35 | 60.68 | 49.29 | -7.34 |
| 30 | hsa-miR-422a | 60.00 | 61.00 | 38.10 | 79.22 | 66.00 | 60.71 | 17.91 |
